# Minutes and Discusions of the Indiana State Dental Convention

**Published:** 1859-03

**Authors:** 


### Proceedings of Societies.

### MINUTES AND DISCUSIONS OF THE INDIANA STATE DENTAL CONVENTION.

In pursuance of a call made at a preliminary meeting held on the 18th September last, in the city of Indianapolis, a large number of the profession of that, and other States met in convention at Ramsey's Hall, for the purpose of forming a State Dental Association.

The proceedings of the preliminary meeting were read and acted upon in reference to a permanent organization.

Dr. John F. Johnson, who was president of the preliminary organization, delivered an address on the objects of the Association, (which may be seen on another page,) Dr. G. H. Perine, of N. Y., moved that the chair appoint a committee to arrange order of business; Drs. P. G. C. Hunt, Indianapolis; G. Lupton, Shelbyville; A. M. Moore, Lafayette, were apointed by the chair.

Order of Business.—1. President's address; 2. Reading of the minutes; 3. Presenting Constitution and By-Laws; 4. Election of officers; 5. Election of members; 6. Appointing committees; 7. Reading papers and essays; 8. Reports of committees; 9. Discussions.

Subjects for Discussion.—1. Best means of preserving the teeth; 2. Treatment of exposed nerves; 3. The best means to correct irregularity of the teeth; 4. Mechanical dentistry; 5. Filling teeth; 6. Miscellaneous.

P. G. C. Hunt,<br>

G. Lupton,<br>

A. M. Moore, } Committee.

On motion of Dr. A. M. Moore, Resolved, That a vote of thanks be presented to Dr. Johnston, for his interesting address; with a request of a copy for the Dental journals. Adopted.

Dr. G. C. North moved that a committee of five be appointed to prepare Constitution and By-Laws.

The following were appointed by the chair:

G. C. North, Indianapolis,
P. G. C. Hunt, do
R. H. Hurd, Attica,
S. B. Smith, Terre Haute,
G. Lupton, Shelbyville,
} Committee.

On motion of Dr. Satterwite, of Franklin, Resolved, That the Constitution be received and the Committee discharged.

On motion of Dr. P. G. C. Hunt, the Constitution was adopted and the association entitled the Indiana State Dental Association.

CONSTITUTION OF THE INDIANA STATE ASSOCIATION.

*Article* 1.—This Society shall be known by the name of the Indiana State Dental Association.

*Article* 2, *Sec.* 1*st.*—The officers of the Society shall consist of a President, three Vice Presidents, a Recording and Corresponding Secretary, Examining Committee of five and Treasurer.

*Sec.* 2*d.*—The officers shall be elected by ballot, at each annual meeting, and their duties shall be such as appropriately belong to such officers in other deliberative bodies, a majority of votes only shall be necessary to a choice.

*Sec.* 3*d.*—The association now forming shall be chosen from members of this convention, by ballot, two thirds of all the votes given being necessary to an election.

*Sec.* 4*th.*—After the formation of this Society, all applicants for membership shall be examined by the examining committee, and whereas, the chief objects in organizing this association, is the elevation of the profession in Indiana, and to promote harmony and concert of action among the *legitimate* thereof, to promote the free interchange of sentiments both written and verbal between members of this association, and to draw a line of distinction between the worthy and unworthy. Therefore, Resolved, that no one shall be eligible to membership unless he possesses a good moral character, a legitimate practice which shall be understood to mean the practice of dentistry to the exclusion of all other callings, neither must he resort to the empirical practice of self-laudation in newspapers, or any other undue means:—this to apply to the reception of members during the formation of this association, as well as afterwards.

*Sec. 5th.*—All candidates for membership, recommended by the examining committee, shall have (in order to be received as members,) sustained a satisfactory examination on all the branches of Dental Science, and in every other particular, as set forth in Article 4th, provided, however, that those who have Diplomas from a respectable Dental Association may be received without examination by the committee.

Applicants recommended by the committee shall be balloted for; two thirds of the votes given shall be necessary for membership.

*Article* 6.—Any member may be expelled from the association for immoral conduct, malpractice, or other sufficient cause, on motion of an investigation committee, appointed for that purpose, by a two third vote of all the members present.

*Article* 7.—This association shall be composed of two classes, viz.:—Acting and honorary members.

*Article* 8.—Each member at his initiation shall pay into the treasury of the society the sum of $3, and annually thereafter the sum of $2, and all those who shall fail for two successive years, to pay their annual dues, shall forfeit their membership.

*Article* 9.—No member of this society shall take a student for a shorter period than two years, unless he shall have received instructions previously to equal the term.

*Article* 10.—The meetings of this society shall be held semi-annually, at such time and place as a majority of the members present at each, may determine.

*Article* 11.—The association may receive contributions in money, books, or other property, which may be either used or sold in aid of its purposes.

*Article* 12.—Seven acting members beside the president and chairman shall be necessary for the transaction of business, at the opening of any meeting.

*Article* 13.—In the event that a quorum can not be obtained at any regular meeting of the association, the time and place on the following year, shall be the same as on the preceding year.

The president shall have the power in case of a failure of any meeting, to call a meeting of the association at any time that he may deem proper.

*Article* 14.—This Constitution may be amended by a vote of two thirds of the members present at any regular meeting of the association.

On motion of Dr. Perine, the election of officers was proceeded with, which resulted as follows:

The chair appointed as tellers, Drs. G. H. Perine of New York City, John T. Toland, Cincinnati, Ohio.

*President.*—Dr. J. F. Johnston, Indianapolis.

*Vice Presidents.*—Drs. J. P. Ulrey, Rising Sun; P. G. C. Hunt, Indianapolis; A. M. Moore, Lafayette.

*Recording and Corresponding Secretary.*—Dr. G. C. North, Indianapolis.

*Treasurer.*—Dr. T. M. Nichols, Indianapolis.

*Examining Committee.*—Drs. H. R. Hurd, Attica; J. Wood, Greensburg; P. G. C. Hunt. Indianapolis; J. P. Ulrey, Rising Sun; S. B. Smith, Terre Haute.

On Motion of Dr. G. Lupton: Resolved, That we admit no dentist to membership in this society, who has not a fixed place of residence, or who is in the habit of moving from place to place, to avoid the responsibility of his quackery, thereby bringing reproach upon the profession, generally. Adopted.

Election of members was next in order, and Constitution signed.

Drs. G. H. Perine, of N. Y. City; J. L. Broffett, Paris, Ohio; John T. Toland, Cincinnati, were voted as honorary members of the association.

On motion by Dr. Hunt, adjourned to meet at 7 o'clock.

Evening Session.—President in the chair. Minutes read and approved.

The Secretary read a letter received from J. Hass, of Lafayette, Indiana.

On Motion of Dr. Perine, the letter was received.

Dr. H. R. Hurd, of Attica, presented the following resolution, which was read and adopted:

*Resolved,* That the Medical and Dental profession in good standing from abroad be invited to participate in the discussions.

The 1st subject, Best means of preserving the teeth was presented for discussion. Drs. Hunt, Moore, Nichols, Hurd, Smith, Perine, North and others expressed their views.

On motion, adjourned to meet at 10 o'clock, A. M.

SECOND DAY—MORNING SESSION.

Minutes read and approved.

The following resolutions were presented:

By Dr. G. R. North, *Resolved,* That the Treasurer be directed to pay all orders for monies belonging to the Association which are signed by the President and Secretary thereof.

By Dr. Lupton, *Resolved,* That we now consider the time and place for next meeting. Adopted.

*Dr. Lupton* moved, That when we adjourn, we adjourn to meet at Shelbyville, Ind.

*Dr. Hunt* moved to amend it by substituting Indianapolis.

*Dr. North* moved, that the resolution be laid on the table. Carried.

*Dr. North* offered the following, which was adopted: Resolved, that the clause in the Constitution be altered from semi-annual to annual meeting.

On motion, it was unanimously resolvod, that when we adjourn, we adjourn to meet in Indianapolis, Ind., on the first Tuesday of January, 1860.

*Dr. G. H. Perine* of New York, presented the following resolutions of condolence on the death of Dr. Elisha Townsend of the Philadelphia Dental College, which were unanimously adopted.

*Whereas,* It has pleased an all-wise Providence to remove from among us our much esteemed, and highly honored fellow member, Dr. Elisha Townsend, Be it therefore

*Resolved,* That it was with sincere and painful regret that we received information of the death of this worthy member and shining luminary of our profession—whose talents and learning, and devotion to science; and whose sincerity, and unselfish liberality endered him to all hearts. He was ever foremost in promulgating truth and exposing error. His whole life was a scientific and Christian sacrifice for the adornment of his profession, and his fellow men, and the absence of his aid and counsel will make a void not easily filled.

*Resolved,* That we sympathise with heartfelt sorrow with the family and friends in their affliction, and unite in offering to his memory that tribute of admiration and respect which is due to exalted genius, and devotion to science for the benefit of his fellow men, and the elevation of the profession to which he belonged and loved.

*Resolved*, That the Secretary be instructed to send a copy of these resolution to the bereaved family, and the Dental Journals for publication.

Treatment of exposed nerves being the next subject in order, a paper was read by the President from Dr. W. H. Shadoan, of Rockport (which see another page), which was afterward discussed by Drs. Smith of Terre Haute, Hunt of Indianapolis, Hood of Greensburg, Lupton of Shelbyville, Satterwite of Franklin, Moore of Lafayette, and Baraffett of New Paris, Ohio.

After the discussion closed, on motion by Dr. North, Resolved, That a committee of three be appointed to prepare the order of business for the next meeting, and furnish copy early to the Dental Journals for publication, and recommend that the profession come fully prepared.

Drs. J. P. Ulrey, Rising Sun; A. M. Moore, Lafayette, and W. R. Webster were appinted.

By Dr. Lupton, Resolved, That a committee consisting of three be appointed to draft and present a Fee Bill for adoption.

Committee—Drs. G. Lupton, Shelbyville; R. H. Hurd, Attica; T. M. Nichols, Indianapolis.

The third subject was now presented for discussion, "The best means of correcting irregulartity of the teeth."

The subject was discussed at some length, by Drs. Moore, Dills, Johnston, Hood and others.

On motion, the subject closed, and the fourth subject—Mechanical Dentistry was taken up and discussed by Drs. Satterwite, Perine, Hunt, Moore, Johnston, Hood, Nichols and others.

On motion, adjourned to meet at two o'clock.

AFTERNOON SESSION.—On motion of Dr. Smith, the reading of the minutes be dispensed with. Adopted.

The last subject under discussion was continued, by request of Dr. Hunt; it was resumed by Dr. Perine, of New York, and discussion continued by Dr. Hurd and others.

A letter was read from Dr. Shadrun, regretting his inability to be present, and promising future co-operation. On motion the letter be received and placed on file.

Subject 5th—Filling Teeth was then taken taken up and discussed by Drs. Hunt, Satterwite, Perine and others.

On motion of Dr. Hunt, adjourned to meet at 7 o'clock, to discuss miscellaneous matters. Adopted.

EVENING SESSION.—Association met pursuant to adjournment. Minutes of preceding meeting read and approved.

*Dr. Lupton*, chairman of the committee, on Fee Bill, read report.

On motion, report was received and committee discharged.

*Dr. Hunt*, moved to take up Fee Bill, examine item by item, modify, reject, adopt, and recommend as a whole to the next meeting of the association.

As follows—adopted.

| | |
|---|---|
| Full set of Teeth on Gold or Platina.................... | $100 to $150 |
| One Tooth, on Gold Atmospheric pressure.............. | 10 " 12 |
| Two Teeth, " " " .............. | 12 " 25 |
| One Tooth, " With Clasps.......................... | 8 " 10 |
| Two Teeth, " " " .......................... | 10 " 12 |
| Three Teeth, " " " .......................... | 15 " 18 |
| Four to ten " " " $4 to $6 per tooth. | |
| Obturator................................................ | 50 " 100 |
| Filling Molar Tooth (Fang included).................... | 12 " 25 |
| " Incisor or Bicuspid " .................... | 6 " 15 |
| All other filling............................................ | 2 " 10 |
| Extracting teeth in office (each)........................ | 50 cts. to $1 |
| Visit extracting or advice................................. | 1 to 5 |
| Administering Chloroform................................. | 2 to 15 |

On motion of Dr. Hunt, adjourned to meet at 7 o'clock, to take up miscellaneous business.

Miscellaneous subjects being now in order, were discussed by Drs. Hunt, Nichols, Johnston, Smith, Perine and others —interesting cases and specimens were presented.

On motion of Dr. Hunt, association adjourned to meet on the first Tuesday in January, in the city of Indianapolis, Ind., 1860.

Pursuant to previous invitation, (extended to the entire association) upon the adjournment of the association, some fifteen or sixteen of the Dental, with several friends of the medical profession, repaired to the residence of Dr. J. F. Johnston, (our worthy President) to partake of his hospitalities.

At 9 o'clock, all sat down to a table most bountifully supplied with all the delicacies which could tempt the palate, and to witness the performance would convince an observer that—however self-denying in other respects, in this case, they could not resist temptation.

After partaking of the good things set before us, the President, Dr. Johnston, made a neat and pointed address, expressing his pleasure in meeting the profession in convention in Indianapolis, particulorly in meeting them at the same board, under his own roof.

The following sentiments were presented by Dr. Perine:

The Medical and Dental professions. In union is strength; may they ever be equal to the most obstinate *molar*, and in unity as beautiful as the most perfect *Continuous Gum.*

Responded to by Dr. Parvin, of Indianapolis, who spoke of the relationship of the professions, and the necessity of the same for the general good of mankind.

By Dr. Lupton—The Medical profession, may they always be ("Wright.")

Dr. Wright, of Indianapolis, responded and gave the following: The Dentist, a Scarecrow, used by indulgent mammas, to frighten unruly children.

Beautifully responded to by Ladies present.

By Dr. North—Our hostess. May she live to receive the reward for her present kindness in the memory of the profession, when the present members have given way to a more permanent Set.

Responded to by Mrs. J. F. Johnston, expressing herself delighted.

By Dr. Hunt—The Dental Reporter, may he soon be able to report her.

Toland, responded as follows:

Though too modest to attempt to do justice in behalf of our amiable hostess, and not a ladies' man; still having been called upon, I deem it imperative to serve. Actions are but the reflex of the mind and heart. From brief acquaintance and observation of her kindness and hospitality, I feel warranted in the assertion that Mrs. Johnston seeks no greater happiness than what is afforded by the appearance of enjoyment she has been enabled to afford her guests now present. Generosity and liberality are the twin sisters of that noblest quality—"mercy"—which as the poet describes it,—"Falleth as the gentle dews of Heaven, upon the place beneath, it is twice blessed, it blesseth him who gives and him who takes; 'tis mightiest in the mightiest." May the organization of the present meeting of the Profession receive cultivation to insure the full and perfect development of an arch, which may permanently bind together succeeding generations of Dentists to their scientific elevation, and the alleviation of all the ills to which the Dental organism is subject.

Dr. MOORE—While we are favored with a (Hunt,) may we often meet with a Hurd.

Dr. HURD—May the herd of Dentists ever enjoy themselves as we do now.

By Dr. JOHNSTON—Our Brethren at the East.

Dr. PERINE being called up, responded as follows:

Mr. President—It is with pleasure as well as with diffidence, that I respond. It is with pleasure, Mr. President, that I look upon the faces of those with whom agreeable reminiscences are associated. Pleasant to meet where many of us for the first time meet each other—pleasant to see the members of our profession, and it is pleasant to be among you, to form new and renew old acquaintances, and to bring to recollection days gone by.

It is by the co-operation of such of my professional brethren as I see before me and through the association of the press and our schools, the good influence has spread over the length and breadth of the land, from the Shores of the Atlantic to the Coasts of the Pacific its influence has telegraphed its way. Our Institutions founded by such skill and reared by such labor must endure. Hereafter we may look forward to the increased and steady advancement of our profession: long may the influence be felt in the hearts of the brethren, and when generations shall have passed away, let the representatives of this and other similar Associations unite in their Yearly Anniversaries as we do to-day, ever improving and improved.

At a late hour all retired delighted and satisfied with the entertainment, as well as the labors and achievements of the association, a more cordial and enthusiastic spirit seemed to pervade (all feeling that it was good to be here), determined to push forward the objects of the association with a hearty good will.

### ADDRESS OF DR. J. F. JOHNSTON.

GENTLEMEN: As chairman of the preliminary meeting, which adjourned as you have heard to meet here to-day, it is made my duty to call this body to order, and preside over it until the regular officers are elected. I must ask your kind indulgence however, for the many errors I shall undoubtedly commit even during my very brief term of office, for I am mainly unaccustomed to such duties. I have also been requested to make a few remarks with reference to our undertaking. This, gentlemen, it would almost be needless to tell you, I am *entirely* unaccustomed to. I am a Dentist, and *not a talking man*, I therefore indulge the hope, that you will not expect much of me, and will bear kindly with my imperfections.

It gives me great pleasure to see as many of the profession in attendance on this their first general meeting, that I am aware of that ever took place in the State of Indiana, as are here to-day. I trust it will be far from being the last time that we shall have the pleasure of meeting each other under similar circumstances. And now, allow me on the part of those Dentists in this city, who have felt an interest in this movement to bid you a cordial and hearty welcome! Gentlemen, we are *glad* to see you. The circular which all of you have seen, either copied into the Dental Journals, or in its original form, calling this convention, explains in the main its object. It will be seen that we have met for the purpose of aiding in the accomplishment of a good work, for surely the elevation of our profession, the cultivation of friendship, and good will towards each other, mutual improvement, and the crushing out of empiricism, and as a result of all this to benefit our patients, and indeed, the community at large, may be with all propriety so considered. Then with this in view, should we not enlist with zeal, and use our best talents in so good a cause, will not all then enter into the business of the convention, with kindly feelings, and a determination to contribute what they can, for the general good, and assist heartily in pushing matters forward to a successful and satisfactory completion. It is well known to all of you, that in many of the States of this Union, our professional brethren, have gone energetically to work in the formation of societies, either State or local. The result is as you are aware, not only a pleasant acqaintance with each other, but a decided advancement in professional attainments, the beneficial effects of which even we in Indiana, can not help but acknowledge. For example, look what our professional brethren in Ohio, are doing with their Northern association, their Ohio college association, and more recently the Cincinnati association, as well as the Mississippi Valley association, which always I believe has met in Cincinnati. Gentlemen, they have learned the advantages to be derived from these meetings, and consequently like sensible men they keep them up. But we have said nothing of the New York, Pennsylvania, Michigan, Illinois, and other societies, which are "all alive," and making themselves useful. Should we then in Indiana, who you see are almost surrounded by institutions of this character remain inactive recipients of the benefits they confer, without making any exertions to contribute to the general fund? Or, to speak more plainly, should we remain isolated and selfish, warring upon each other with but one idea, and that a very stale one too—*money*—or should we combine, fraternize, and with our united talents, help to fill the measure of dental science with something of *value* from the land of "Hoosierdom?"

We have many good practical men in Indiana, who if they would but put their shoulder to the wheel, we should not have to wait long "for the wagon," it would soon roll along well loaded with valuable facts, for the profession. It is with a view to bring out such men, promote harmony, improve each other and kick out quackery, that we have ardently desired the formation of this association. I have long entertained this feeling, have indeed been talking of it and advising with a few of my professional friends for the past few years, and on further consultation with a few friends at the American Convention, and afterwards with some of my professional brethren here, it was determined to put "the ball in motion." Gentlemen, you see that ball in motion now, we hope you will all feel bound to give it a push onward. Let us get up the long wished-for perpetual motion that will work the brains and the muscles of all our Dentists in the State to a good purpose. I can very well recollect when dentistry was away "below par," when compared with other professions, all of you must at least have noticed a change for the better each succeeding year you have been in practice. A Dentist to succeed now must be something more than a mere mountebank. To what are we to attribute these improvements? Gentlemen, it is the formation of societies similar to this we have now in view, but more particularly however to the healthy influence of our Dental Colleges and Journals.

Permit me, gentlemen, to digress for a few moments to say a word or so about them. I feel that our Colleges must be sustained and I trust all of you will urge your students to pursue a regular and legitimate course of study.

The formation of these societies will in my opinion eventually do more for our colleges than any thing else. They have a tendency to excite the ambition of the student, he will desire to be even with his neighbors, he will thirst for a thorough knowledge of his profession, and it is not for a moment to be doubted, where he will go to get it. I am well aware that many of our "Stars" in the profession (if you will allow me to use this theatrical phrase), did not have the opportunity of enjoying the advantages of our Dental Schools, many of them, indeed, being in active practice long before they were established. They have succeeded through unwavering perseverance, and their natural talents. Yet no doubt most of those men, if they had had the advantages which are within the reach of students at the present time, would have gladly availed themselves of them, in preference to taking the hard road which they must have had to travel. These very men indeed, appreciating these difficulties, established our colleges. We of this day should ever hold them in kind remembrance for it.

But, gentlemen, we must not forget the benefits received through the medium of our periodicals, which have undoubtedly done more for the dental profession than any other one thing, they being the means by which information in a tangible form has been so widely and thoroughly diffused. They have been the means of raising many, out of "the mire and the clay." To them must be attributed the formation of these societies, and even our colleges; for those pioneers of whom I have been speaking made themselves heard through their instrumentality; in fact, they have placed dentistry where its most ardent friends thirty years ago never even dreamed it would be, viz: on a respectable footing with the other professions. I think it is therefore the duty of each and every one who has the interest of his profession at heart to subscribe and *pay for*, at least one of our standard Dental Journals, and not content himself because he is in the occasional receipt of some advertising medium, that may have a page or two of borrowed matter in it; or if he does so content himself, he must not suppose he is doing his whole duty as a member of a progressive profession like ours. No profession will progress unless its literature is encouraged; on the contrary it will retrograde. We have in ours several good journals, edited with ability, for the good of the *profession*, and not to advertise; they have done, and are yet doing good, are disseminating a vast amount of useful information, and they ought to be liberally and cheerfully supported. The others can take care of themselves. It is unfair to suppose that men of ability can devote their time to the editing of a dental journal, just for the notoriety of the thing or through motives of philanthropy. They eat and wear clothes as other men, and so do their families (and I believe they generally have them, for they seem to believe in the propagation of ideas in every conceivable form.) Then, when we acknowledge, as we must, the benefits they confer, it is easy to see that we ought to give them support.

But seriously, gentlemen, my reasons for calling your attention so particularly to this point, are these: There is such an increase in these advertising periodicals, which are scattered almost gratuitously over the office of every Dentist, that I have felt some alarm that the profession might lose sight of the advantages we derive as a profession, from our standard Journals, (from which, indeed, those others receive the chief part of their mental pabulum,) and withhold their patronage; when of course they would have to wind up, and we should again be left in professional darkness; for I must again repeat, that although a Society of Dentists first led to the publication of the first dental Journal, yet after that journal, and others were in operation, they did more towards the formation of such societies, and the elevation of the profession as a mass, than any thing else. And now, I trust, I shall not be misunderstood as warring upon these journals, that are published by individuals, to extend their own business and interests, they certainly have a most perfect right to advertise in whatever manner may seem to them best. I consequently can have no objection to their publishing these works, but I wish to impress upon the profession the necessity of liberally sustaining those works which are devoted to its interests only—that it may be enabled to keep its present fair position in the eyes of the world, as an honorable profession. So gentlemen, let us lend a helping hand in all these good works, by sustaining our societies, and requiring our students to sustain our colleges, and ourselves support our dental Journals, and you will soon see quackery on the wing to a more congenial climate; for the intelligent dentist gradually but surely educates the community in which he he lives, as to what they may reasonably expect of an accomplished operator. They soon learn that certain things can be successfully done if properly managed, and will consequently not be satisfied with what a quack would be able to accomplish; for in our profession, as all are aware, it is much easier to exhibit our skill, or our *awkwardness and ignorance*, than in most others. Our friends will excuse us if we institute a comparison with them, we being relations in as much as we belong to that ever-increasing family, the Doctors. Now, with them there is a much better chance for a quack than with us, "dead men" you know, "tell no tales." But with us deformed mouths, marred expressions, defective plugs, and broken jaws, tell a great many—they all most emphatically "speak for themselves," and point so unerringly to the quack, that he can not be mistaken. So as I remarked before, let the profession unite for the common good, and humbug dentists will have to improve or vanish. I know of no better way for us to unite, than has already been proposed, by the formation of the Indiana State Dental Association. I therefore hope, we shall be able to congratulate each other upon the successful accomplishment of this object. I can only add, gentlemen, that I ardently desire to see it vigorously sustained, and trust that at our next regular meeting, we shall appear in greater numbers than at the present.

But, gentlemen, I will not occupy the attention of the convention longer, for we have much of general interest to attend to. I have talked about matters that would seem to be foreign from our present subject. I can only apologize by saying, the intention was good. I thank you for your attention.

### PAPER READ BY DR. W. H. SHADOAN.

Mr. President: I desire, in as brief a manner as practicable, to give my process of the treatment of exposed nerves of the teeth.

We will take, if you please, the first bicuspid of the right side of the superior maxilla. The dentine is decayed so as to leave the nerve exposed but not injured. It seems to be in a healthy state, so far as the indications show.

The first thing to be done is to carefully remove the diseased bone surrounding the nerve, and also all the diseased parts of the tooth in question. After which, I take a shot (a shot which has been kept in a dry place is preferred), beat it out thin, say about No. 30 American plate gage. Cut a piece as large as will cover the nerve, and overlap the dentine sufficiently to form a hold. I then carefully lay the cap so formed, on the nerve, carefully press it to the tooth around the nerve. And then proceed to fill with gold foil. In all such cases I use what I call block filling. My mode of preparing my blocks are by folding my gold so as to make it four double, cut the piece into three strips, one of which is as wide as both the others. I then roll them together very loosely, and cut off my blocks of the smaller roll, and commence by laying one on one side of my cap so as to overlap it about one-eighth of the size of the block, then treat the opposite side in the same way. Then begin on the ends (as we will call them) lay one of your blocks on the posterior part of the cavity, let it lap upon the two former blocks as on the cap, press it sufficiently hard to make it retain its position. Treat the anterior block as the posterior. Then take a block of the larger roll, and lay on the center cavity, and condense slightly. This as you see has formed an arch over the cap, so that the remainder of the process of manipulation will not affect the nerve by pressure. After it is thus far successfully filled you will proceed by filling around the arch. Always pressing at right angles with the bottom of your cavity. By carefully following this rule you will find that your filling will always incline towards the walls of the cavity, and the consequence will be the plug will not want to dislodge itself while you are building it up. Besides, this arch that is formed over the cap assisted by the cap, keeps the nerve from being injured by under pressure. I find that I can make as hard a filling in this way as though the nerve were not exposed.

In case the nerve is diseased your success will depend upon your diagnosis. If nothing but inflammation has set in you can remove that by treating your patient constitutionally. If my patient needs it, I give a cathartic and apply a little creosote, or tannin, [I almost always use tannin, or sulphate of zinc, and in case the tooth is very sore, I will take tannin and morphia in equal parts, and find that it is just as good treatment as I can adopt.

In case the destruction of the nerve is necessary, I accomplish that by the application of metallic arsenic and creosote, which in nine cases out of ten is successful; if it should fail, I then use the potential cautery, such as nitrate of silver, or most generally nitric acid, and in the event of their all failing, I then extract the nerve in toto. I then carefully prepare my nerve cavity, and fill with pure gold, after which I hear no complaint. I find the nerves of teeth diseased when there is no decay in the teeth. In such case I use the drill freely until I have formed a cavity sufficiently large to admit of my operation, then I extract the nerve and syringe the cavity well with creosote, tinct. opii, chloroform and acconite. Then I plug the cavity, and hear no more complaint from that source.

DISCUSSION OF SUBJECTS.

1. *Best means of preserving the Teeth.*—Dr. HUNT said, when the teeth were in a healthy condition he would recommend care, and frequent use of the brush and soap. When teeth are diseased, and require filling, he uses gold, and has been successful with the most simple treatment. With children he would recommend the frequent use of a napkin to create gentle friction.

Dr. MOORE (Lafayette), would prefer to listen to older members on the subject. He advised cleanliness, and thinks much depends on the general health of the patient. Thinks in many cases *caries* is hereditary. In peculiar reddish decay, has found that filling would not arrest it. Is of opinion that teeth decay more rapid during the period of pregnancy.

Dr. NICHOLLS asked, in cases of acid secretions, would it not be proper to correct? It might be done with carb. soda, lime, etc., to improve the glandular system; such cases take time. In relation to *caries*, has only discovered three kinds; white caries being the most difficult to arrest. He related the experience of an old practitioner, who, after removing all decay, cut and drilled away the sound bone beyond decay; and thus had no more difficulty in preserving than other kinds of caries. He recommended the use of a soft brush; a hard brush, he thought would wear the enamel into furrows. Nature would not repair the damage done by a hard brush. Recommended cold water, and no dentifrice. He doubted if caries after occurred but from irregularity—teeth being crowded. Mechanical pressure made the enamel thin between the teeth, which are more liable to decay.

Dr. Hurd (Attica), considered cleanliness of the highest importance, no matter how attained. Related experience with some healthy teeth, well developed; the spaces between the molar teeth admitted lodgment of food, which caused decay, before attention was paid to removal, etc. The next case was that of a child. Gave personal and diligent care to cleansing the desiduous teeth, and filled as decay presented itself; they were at present in good condition, and thinks they have been preserved longer than they otherwise could be. He hoped all would engage to throw light to aid each other to benefit patients.

Dr. Smith (Terre Haute), said, that in considering the subject it was necessary to inquire the cause of decay. He would say, want of perfect cleanliness was one of the causes. He thought when the chemical agents were brought to bear upon the surface of the tooth, in nine out of ten cases the causes are external, or brought about by the influence of acids causing receding of the gum where the tooth is more easily acted upon. He differed from Dr. Nicholls in the use of a brush, but would not use brickbats. Thought the teeth could not be injured by the use of a stiff brush and good dentifrice.

Dr. Hood (Greensburgh), said he was satisfied that caries occurs from other than external causes. He referred to cases on cutting edges, where the orifice was very small, when on opening with the excavator, he was astonished at the extent of decay beneath. He thought it resulted from defect in elements which constitute the teeth, and from deviation from some natural laws.

Dr. Perine (N. Y.), on being called upon, said, he had come like a dry sponge, and expected to absorb much, and he was willing to be squeezed. This subject carried them back to the question: How shall we marry? How shall we rear our children? We must go back to the mother from the moment of conception. He was favorably impressed with the use of phosphate of lime, and earnestly called attention to its administration. If decay occurred the teeth should be promptly filled. Cleanliness, of course, was of the utmost importance.

Dr. NORTH (Indianapolis), concurred with the suggestions made in regard to preservation of the teeth. Some diversity of opinion appears. Dr. Perine adheres to the doctrine of Harris, and he inclined to the same. He was aware that a more modern argument was advocated, viz: that the causes of decay are entirely external. He was not satisfied which; whether external or hereditary. He would like to have the matter determined. Recommended frequent use of brush, even at a very early age.

Dr. NICHOLLS remarked, in reference to decay—large cavities with small orifices—Might it not occur by teeth being worn down, until cold is carried to the pulp? Would not this wearing finally go on until the nerve was destroyed? In such the dry kind of decay is discovered; nature seeks to throw off the dead substance. Dr. Perine, he said, had covered the ground. That diseases of the teeth are in some cases constitutional there can be no doubt.

SECOND DAY—*Morning Session.*

2. *Treatment of exposed Nerves.*—Dr. SMITH remarked on the importance of the subject. He thought a large proportion of teeth coming before them with exposed nerves could be saved.

Dr. NORTH said he had given much attention to the subject. He cleanses the cavity free from all carious matter, guarding the nerve from wound or injury, and uses prepared anodynes, antiseptics, etc., to bring about health to the parts. Next prepares cavity for permanent filling by caps, with confidence; he can not leave too much space under cap. He succeeds in eight cases out of ten.

Dr. HUNT said he would like the subject discussed by question and answer, to elicit the experience of all. He has had poor success in capping exposed nerves; has lost about the proportion saved by Dr. North; prefers to remove if the nerve has been wounded. He inquired of Dr. North, when the nerve had been capped, and the filling removed, if any deposit of bone or dentine was found?

Dr. NORTH said he had not had occasion recently to examine.

Hr. HUNT said he had examined many such cases, and found no osseous deposit when the nerve had been wounded, but frequently the death of the nerve.

Dr. HOOD said he had used a variety of preparations in the treatment of exposed nerve, and a variety of articles for capping nerves—bone, lead, etc. His patients were not willing to pay for the treatment of filling roots. In many cases patients were satisfied for one or two years with nerves capped, but not much longer. Had found no bony deposit under caps when teeth were removed, contrary to expectation, but nerves dead, and frequently slight decay in progress.

Dr. HUNT asked if he treated all patients alike who applied?

Dr. HOOD replied, he discriminated of course. In some cases would not treat; preferred not to do so.

Dr. LUPTON (Shelbyville), thought it important to report failures, as they learned more by them than from success. He had more trouble with young patients, and more failures. He found it difficult in the country to get his patients to visit him promptly. Destroys nerves with arsenic, morphine, etc.

Dr. NORTH had two cases to report of failure. First, nerve capped—was obliged to remove filling; treated, destroyed, and filled fang, successfully. The second was that of a young lady, sixteen years of age. External caries slight. On opening and excavating cavity, found nerve exposed; capped and filled with amalgam; it became painful. He then removed the filling, and found the cap too small, the filling pressing upon the nerve. After treatment recapped and filled, but again failed. He again removed the plug, and destroyed the nerve, and is now preparing to fill the root. He never destroys nerves where he can cap and fill.

Dr. SATTERWHITE (Franklin), asked members what they did when the cavity was so shaped, and the nerve so exposed that it could not be protected by a cap?

Dr. JOHNSTON replied that he had used with much satisfaction Plaster of Paris as a cap, and for such cases thought it would answer a good purpose.

Dr. HURD thought much depended on the health of the patient. He could not treat all cases alike. Has had success, and many failures, in capping nerves. Continues to treat the same, and is not disheartened by failures. He was successful in one-half his cases, and should encourage to greater efforts. He cited some interesting cases.

Dr. Smith asked what proportion of those cases in which abscess finally occurred could be considered successful?

Dr. Hood thought if the apparently successful cases of capped nerves were examined, the majority would be found dead.

Dr. North thought that few of those cases were successful; they were generally failures. Uses tannin and creoscte, after using chloroform, with much satisfaction.

Dr. Smith remarked he did not think vitality could be preserved in every case. In front teeth did not hesitate to treat and cap, but in back teeth trouble generally ensues. For inflamed nerves he uses caustic, etc.

Dr. Hurd asked if it would be well to attempt treating a nerve for a patient at a distance?

Dr. Smith said he would not hesitate.

Dr. Hurd did not consider distance an objection. He thought the preservation of a tooth worth several trips of twenty to fifty miles.

Dr. North concurred, but left the matter to the discretion of the patient. He generally acted according to the circumstances of the patient.

Dr. Moore treated healthy nerves only. Uses chlo. zinc, and treats but few, and those of the most favorable cases. Would not allow an ulcerated tooth to remain in the mouth.

Dr. Johnston thought much of chlo. zinc; though it gave acute pain on application, it lasted but for a moment. He would not remove a filling if the abscess was small, but made an incision over the apex of the root, and treated accordingly, if the root was filled. He thinks the zinc has no equal in cases of inflamed dentine.

Dr. Moore thought it advisable to fill a root immediately after the removal of the nerve. Uses creosote, morphine, etc.

Dr. Bkoffett (Ohio), here made some beautiful and appropriate remarks on thorough physiological and hygenic education and examinations, adding a physiological dissertation on the subject, and remarks, after which the discussion closed.

3. *The best means to correct irregularity of the Teeth.*—Dr. Moore thought this a very important subject. Thought the earlier application was made to the Dentist the better. Described a very interesting case.

Dr. Dills said he liked much the inclined plane in many cases, and recommended cheoplastic as a base. He described two interesting cases in which he had used it satisfactorily.

Dr. Johnston, during the reporter's absence, made some interesting remarks on the subject, which were listened to with interest, and presented casts of an interesting and successful case of irregularity.

4. *Mechanical Dentistry.*—Dr. Satterwhite said for full cases he preferred plaster for impressions; for partial, wax without a cup. He takes the female cast first, direct from plaster model, by placing plaster model inverted, within a rim, and pours metal on it.

Dr. Hunt was in the habit of taking male cast first.

Dr. Moore uses a compound of tin, bismuth and lead for the female cast, after a plan recommended by Dr. Allen.

Dr. Perine said that for full cases he preferred plaster; for partial cases he was satisfied with wax impressions. For partial atmospheric cases he has used a cup struck up of zinc plate, using thin sheet wax quite warm. He prefers for those cases if the gums are healthy and hard, a cut chamber, or, in other words, a double plate; making the chamber only the thickness of the plate. For full dentures he prefers the continuous gum; has used the continuous gum blocks for partial cases with satisfaction. For full dentures, the continuous gum and the rubber were the principal kinds used in New York. He used in some cases partial blocks on gold plate with satisfaction.

Dr. Johnston said he had been using the cut chamber satisfactorily; could not say if it was Cleveland's, Gilbert's, or whose it was; it was the same principle as described by Dr. Fuller at the Convention in Cincinnati.

Dr. Hood had tried many plans, and thought best in no case to let the plate touch the natural teeth.

Dr. Nicholls said he regulated the size and depth of cavity plates according to circumstances.

Dr. Hunt said he also used the cut chamber. First received the idea from Dr. W. H. Hunter, of Cincinnati, who uses double plates; the chamber formed by cutting a hole out of the inside plates, and filling the space between the two plates with continuous gum material.

Afternoon Session.—On call of Dr. Hunt,

Dr. Perine made some remarks on cheoplasty. He said where he came from "the game was played out;" few used it, except for temporary purposes. If he were to use any metal more base than silver, he would prefer pure tin, as recommended by Dr. Geo. E. Hawes some years since, who had been robbed of the credit to which he was entitled. He further said he had seen the work in many mouths, which in some was much discolored and corroded, and in others clean and bright.

Dr. HURD remarked he thought at one time he would try it; he called at the New York Teeth Manufacturing Company to test the matter; offered $20 for the privilege to test it, and if it did not come up to representations the money to be refunded, which was refused; called upon Dr. McDonnell, who thought it was not fully tested, and expected improvements. He said he had not used it, and he did not think he should. Did not like the course pursued with it. Did not like patents in the profession any how.

5. *Filling Teeth.*—Dr. HUNT wished to hear from those who had used adhesive gold foil.

Dr. SATTERWHITE said he had used crystal gold foil, and liked it much; but thought not so much could be put in a cavity.

Dr. PERINE said he was a "blab," especially when a subject was presented which he had made a "hobby," as he had in the use of crystal foil for filling teeth. He said the first thing with him was to make his patients think he was "some punkins" of a Dentist, and he was vain enough to think there were those in New York that though he was "some." He selects a central incisor, and illustrates his operation on the President, as his patient. He first treats the tooth, which he finds considerably diseased; injects the root freely with creosote, and uses nitrate of silver; takes sufficient time in treating, which, when done, two out of three, he thinks, can be saved. Fills root with non-adhesive gold foil rolled into cylinders on fine broach; carried on the broach to apex of root; then with small untempered instruments made for the purpose, after the fashion of the whalebone plugger used by Dr. J. S. Clark, proceeds as he illustrated by cut in the *New York Dental Journal.* Sometimes fills upper roots with Plaster of Paris, injected in the root, absorbing and drying the moisture with bibulous paper and a jet of warm air thrown upon it. If the tooth becomes inflamed, treats with creosote; sometimes applies a leech. When nerve is exposed accidently, prefers to treat mechanically, when he can get the patient to submit.

EVENING SESSION.—Miscellaneous matters were discussed with much interest.

Dr. JOHNSTON exhibited an interesting case of irregularity.

Dr. PERINE presented a beautiful case of continuous gum teeth after the style of work of Dr. B. Franklin, New York.

Dr. SMITH exhibited an interesting specimen where exostosis had been developed.

Dr. HUNT related interesting cases of ossification, his treatment and success.

Dr. NICHOLLS related cases of neuralgia, his treatment and successful results.

Dr. TOLAND presented a neat specimen of vulcanized rubber partial case. He stated that the work was about being introduced into Cincinnati.

